# Molecular epidemiology of hepatitis C virus genotypes in different geographical regions of Chinese mainland and a phylogenetic analysis

**DOI:** 10.1186/s40249-023-01106-y

**Published:** 2023-07-10

**Authors:** Qiao Tang, Zhiwei Chen, Hu Li, Li Zhang, Mingli Peng, Yi Zeng, Xiaoqing Liu, Zubi Liu, Peng Hu

**Affiliations:** 1 Department of Infectious Diseases, Key Laboratory of Molecular Biology for Infectious Diseases (Chinese Ministry of Education), Institute for Viral Hepatitis, The Second Affiliated Hospital, Chongqing Medical University, 74# Linjiang Road, Yuzhong District, Chongqing, 400000 China; 2 Key Laboratory of Digital Technology in Medical Diagnostics of Zhejiang Province, Hangzhou, Zhejiang China; 3 Zhejiang University School of Medicine, Hangzhou, 310000 China

**Keywords:** Hepatitis C virus, Genotype, Distribution, Phylogenetic analysis, China

## Abstract

**Background:**

Hepatitis C virus (HCV) infection remains a major public health problem in Chinese mainland. Investigation of the distribution of genotypes contributed to the prevention, diagnosis and treatment of HCV infection. Therefore, we conducted a study on the distribution of HCV genotypes and phylogenetic analysis to provide an up-to-date understanding of the molecular epidemiology of genotypes in Chinese mainland.

**Methods:**

Our retrospective multicenter study enrolled 11,008 samples collected between August 2018 and July 2019 from 29 provinces/municipalities (Beijing, Hebei, Inner Mongolia, Shanxi, Tianjin, Gansu, Ningxia, Shaanxi, Xinjiang, Heilongjiang, Jilin Liaoning, Henan, Hubei Hunan, Anhui, Fujian, Jiangsu, Jiangxi, Shandong, Shanghai Zhejiang, Guangdong, Guangxi, Hainan, Chongqing, Guizhou, Sichuan and Yunnan). Phylogenetic analysis of each subtype was performed to infer the evolutionary relationship of sequences from diverse regions. Two independent samples *t* tests were used for the comparison of continuous variables, and chi-square tests were used for the comparison of categorical variables.

**Results:**

Four genotypes (1, 2, 3 and 6) were found, including 14 subtypes. HCV genotype 1 was dominant, accounting for 49.2%, followed by genotypes 2, 3 and 6, accounting for 22.4%, 16.4%, and 11.9%, respectively. Additionally, the top five subtypes were 1b, 2a, 3b, 6a and 3a. Proportions of genotypes 1 and 2 decreased while genotypes 3 and 6 increased over past years (*P* < 0.001). Genotypes 3 and 6 were concentrated in the population aged 30 to 50 years, and male carriers had lower proportions of subtypes 1b and 2a than female carriers (*P* < 0.01). Genotypes 3 and 6 were more prevalent in southern parts of Chinese mainland. Nationwide spreads of subtypes 1b and 2a were associated with sequences from northern parts of Chinese mainland, while subtypes 3a, 3b and 6a were associated with sequences from southern parts of Chinese mainland.

**Conclusions:**

HCV subtypes 1b and 2a remained the most common subtypes in Chinese mainland, and their proportions decreased over the past years, while the proportions of genotypes 3 and 6 increased. Our investigation provided an accurate epidemiological picture of the circulating viral strains in Chinese mainland, contributing to the prevention, diagnosis and treatment of HCV infection.

*Trial registration:* Not applicable.

**Supplementary Information:**

The online version contains supplementary material available at 10.1186/s40249-023-01106-y.

## Background

Hepatitis C virus (HCV) is a leading cause of liver cirrhosis and hepatocellular carcinoma [1, 2]. It was estimated that the prevalence of HCV was 0.7% worldwide, with approximately 56.8 million HCV infections. In Chinese mainland, the prevalence of HCV was 0.7% [3]. Data from the Chinese Center for Disease Control and Prevention showed that the incidence of HCV infection in 2021 was 14.38 per 100,000, and with more than 200,000 people infections [4]. This incidence is higher than the new interim target (annual incidence ≤ 5 per 100,000) to eliminate viral hepatitis proposed by the World Health Organization (WHO) [5]. HCV infection remains a major public health problem in Chinese mainland. More effort is needed to accomplish the goal of eliminating viral hepatitis by 2030 [6].

There are 8 HCV genotypes, and many subtypes have been reported. HCV genotype is associated with prognosis and the effect of antiviral treatment [7–11]. Genotype testing is recommended in the guidelines prior to antiviral therapy because genotype is the guidance of treatment [12–14]. The distribution of HCV genotypes varies in different regions worldwide [15]. Genotypes 1, 2 and 3 are distributed around the world, both genotypes 4 and 5 are primarily found in North Africa [16], and genotype 6 is mainly distributed in southeast Asia and southern China [15]. Additionally, the distribution of HCV genotypes varied in diverse geographical areas of Chinese mainland; genotypes 1 and 2 were more prevalent in northern parts of Chinese mainland, while genotypes 3 and 6 were more prevalent in southern parts of Chinese mainland [17, 18]. Strategies for the management of HCV infection should consider the regional distribution of HCV subtypes [19]. Therefore, investigation of the geographical distribution of HCV genotypes is important.

A previous study from 2013 to 2017 showed that the geographic distribution of HCV genotypes was different in diverse regions. Another important finding was that only genotypes 1, 2, 3 and 6 were found in China, and the most prevalent subtypes were subtypes 1b (52.2%), 2a (28.7%), 3b (7.1%), 6a (6.4%) and 3a (4.6%) [17]. However, the proportion of HCV genotypes may have been changed due to the main transmissions of HCV gradually becoming intravenous drug abuse, socioeconomic advancement and migration [19]. Moreover, direct antiviral agents (DAAs) have been available in China since 2017, and the diagnosis and treatment of HCV infection have improved. With an increasing number of patients diagnosed with HCV infection [20], the data after 2017 were closer to the true distribution of HCV genotypes. Therefore, we conducted a study on the distribution of HCV genotypes and phylogenetic analysis to provide an up-to-date understanding of the molecular epidemiology of hepatitis C virus genotypes in different geographical areas of Chinese mainland.

## Method

### Study population and sample collection

This is a nationwide, retrospective, cross-sectional study conducted from August 2018 to July 2019. Individuals were from 29 provinces/municipalities/autonomous regions located in the northern region of China (Beijing, Hebei, Inner Mongolia, Shanxi and Tianjin), the northwestern region of China (Gansu, Ningxia, Shaanxi and Xinjiang), the northeastern region of China (Heilongjiang, Jilin and Liaoning), central China (Henan, Hubei and Hunan), the eastern region of China (Anhui, Fujian, Jiangsu, Jiangxi, Shandong, Shanghai and Zhejiang), the southern region of China (Guangdong, Guangxi and Hainan), and the southwestern region of China (Chongqing, Guizhou, Sichuan and Yunnan). All individuals were screened for serum anti-HCV antibodies for the first time, and 16,806 individuals were positive. Finally, 11,008 patients who were positive for HCV RNA were included in our study, and 5798 individuals were excluded due to negative HCV RNA. The proportion of enrolled patients to the total population of the province/municipality is shown in Additional file 1: Fig. S1. Genotype testing was performed for all serum samples, and a total of 10,751 samples were successfully tested for genotype and subtype, while 257 samples failed to genotype due to the HCV viral load being below 1000 IU/ml. A total of 7796 serum samples were tested for HCV viral load quantification at Hang Zhou Dian Medical Laboratory CO., LTD. The other 3212 patients had already been tested for HCV viral load quantification at the local hospital, and their test results were not available. Genotyping and viral load quantification data were provided by Hang Zhou Dian Medical Laboratory CO., LTD. Additionally, sex, age and geographical information corresponding to the samples were collected.

### TaqMan PCR HCV typing and Sanger sequencing confirmation

The samples were stored at a temperature between 2 and 8 ℃ before detection. The same serum samples were used for the HCV viral load quantification test first and then for the genotype test. If the patient had already been tested for HCV viral load quantification at the local hospital, we did not repeat the test and only performed the genotype test. Two commercially available detection kits approved by the China Food and Drug Administration were chosen to detect the HCV genotypes by following the manufacturer’s instructions. All samples were detected by the Sanger sequencing Diagnostic Kit for Hepatitis C Virus Genotyping (DaAn, Guangzhou, China) to confirm the HCV genotypes or the TIB HCV TaqMan PCR kit (Triplex International Bioscience, Xiamen, China) to detect the HCV 1b, 2a, 6a, 3a and 3b subtypes and mixed infections. The low detection limit of the Diagnostic Kit for Hepatitis C Virus Genotyping is 5000 IU/ml, and that of the TIB HCV TaqMan PCR kit is 1000 IU/ml. The two kits are complementary to each other. If the genotype was not determined by one method, it was tested by the other method, which helped to maximize the successful detection of genotypes.

### Viral load quantification

HCV viral load quantification was performed on a population of 7796 samples using a commercially available kit (HCV Real Time RT‒PCR Kit, liferiver, Shanghai, China) by following the manufacturer’s instructions. The low detection limit of the kit was 25 IU/ml. The other 3212 patients had already been tested for viral load quantification at the local hospital, and their test results were not available. And in this study we used 7796 samples to calculate the mean viral loads of subtypes.

### Phylogenetic analysis

Only serum samples genotyped by first-generation sequencing techniques could provide sequence data for phylogenetic analysis. A total of 2044 partial NS5B partial sequences were available for the construction of phylogenetic trees. The trees were constructed by FastTree 2.1.11 (Lawrence Berkeley National Lab, Berkeley, California, USA) using the maximum likelihood (ML) method algorithm with the General–Time–Reversible (GTR) + gamma nucleotide substitution model [21, 22] and visualized by FigTree version 1.4.4. A bootstrap analysis with 1000 replicates was used to assess the reliability.

### Statistical analysis

Two independent samples *t* tests were used for the comparison of continuous variables, and chi-square tests were used for the comparison of categorical variables. A two-sided *P* value < 0.05 was considered statistically significant. SPSS version 24.0 (IBM Corp., Armonk, NY, USA) was used for data analysis.

## Results

### Distribution of HCV genotypes

**Fig. 1:**
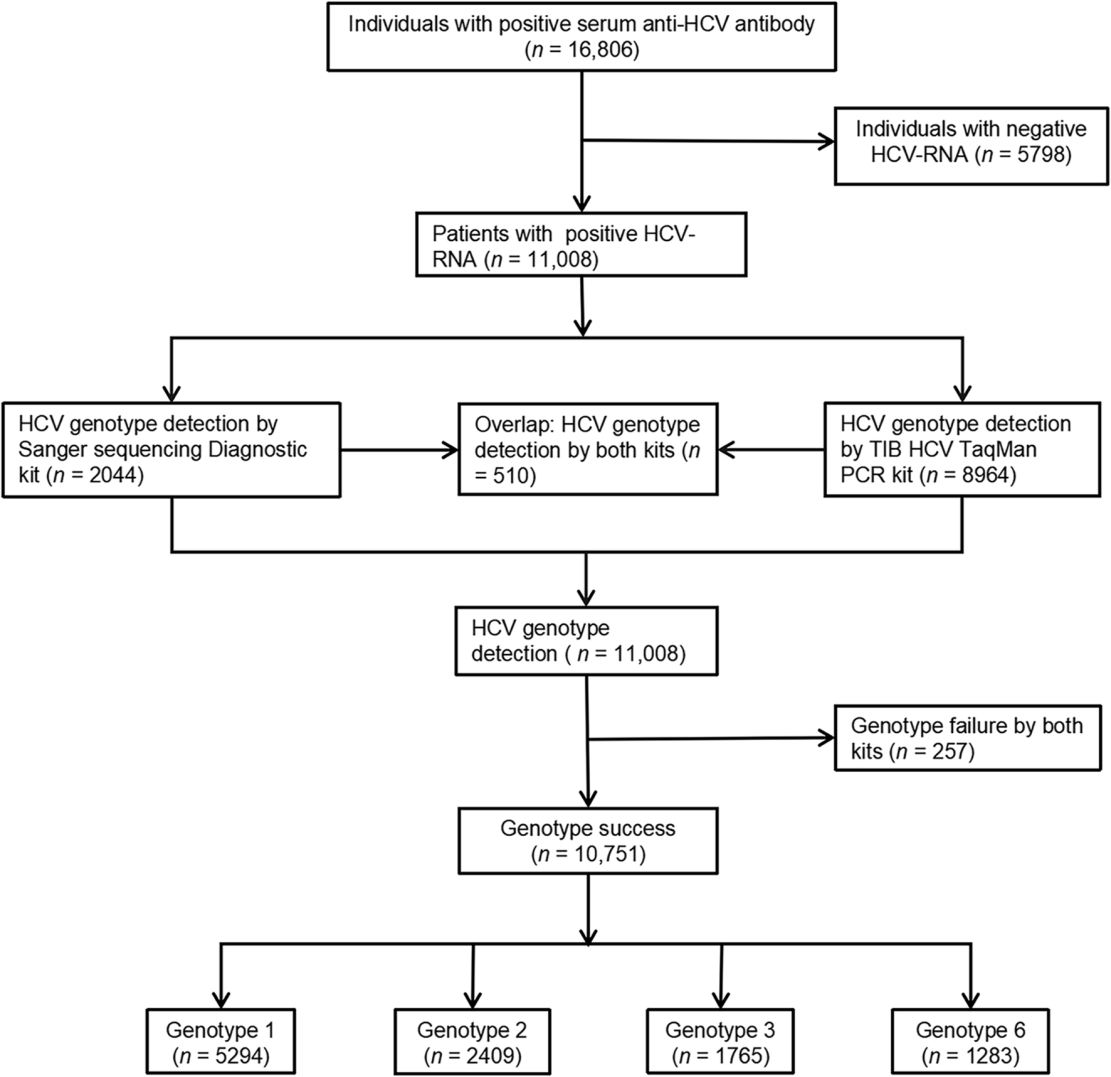
The flowchart of study participants. *HCV* hepatitis C virus, *PCR* polymerase chain reaction, *RNA* ribonucleic acid, *TIB* Triplex International Biosciences

**Fig. 2:**
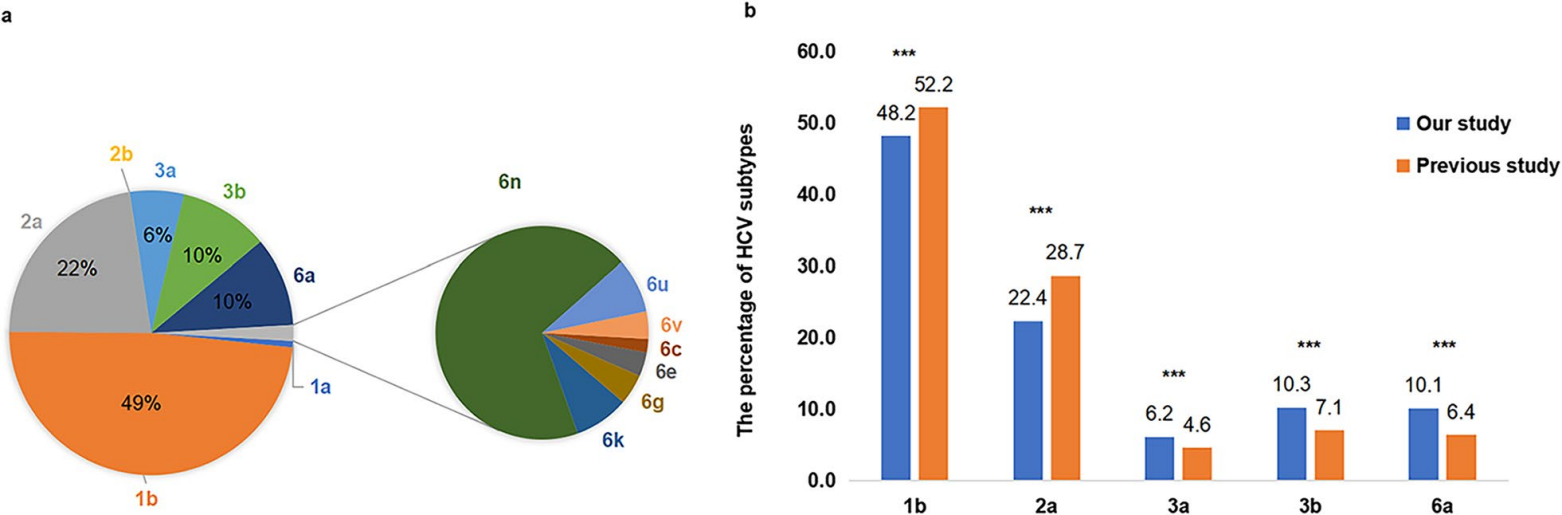
The proportion of HCV subtypes and comparison with previous data. We described the proportion of HCV subtypes (**a**) and made a comparison with previous data from January 2013 to January 2017 (**b**). The horizontal axis represents different HCV subtypes, and the vertical axis represents the percentage of HCV subtypes in two studies. *P* < 0.01 is marked by ** and *P* < 0.001 is marked by ***

Figure 1 shows the flow of the study participants. A total of 10,751 samples were successfully tested for genotype and subtype, while 257 samples failed to genotype. A total of four genotypes (1, 2, 3 and 6) and 14 subtypes were found, while genotypes 4, 5, 7 and 8 were not detected. HCV genotype 1 was dominant, accounting for 49.2% (*n* = 5294), followed by genotypes 2, 3 and 6, accounting for 22.4% (*n* = 2409), 16.4% (*n* = 1765), and 11.9% (*n* = 1283), respectively. Genotype 6 had the most diverse subtypes (6a, 6n, 6k, 6u, 6g 6v 6e and 6c). Additionally, Fig. 2a shows that the top five subtypes were 1b (48.5%, *n* = 5215), 2a (22.4%, *n* = 2406), 3b (10.3%, *n* = 1103), 6a (10.1%, *n* = 1090) and 3a (6.2%, *n* = 662). Figure 2b shows that the proportions of subtypes 1b and 2a were significantly decreased (*P* < 0.001) and subtypes 3a, 3b and 6a were significantly increased (*P* < 0.001) compared to the data from a previous study [17].

### Distribution of HCV subtypes in diverse regions

**Fig. 3:**
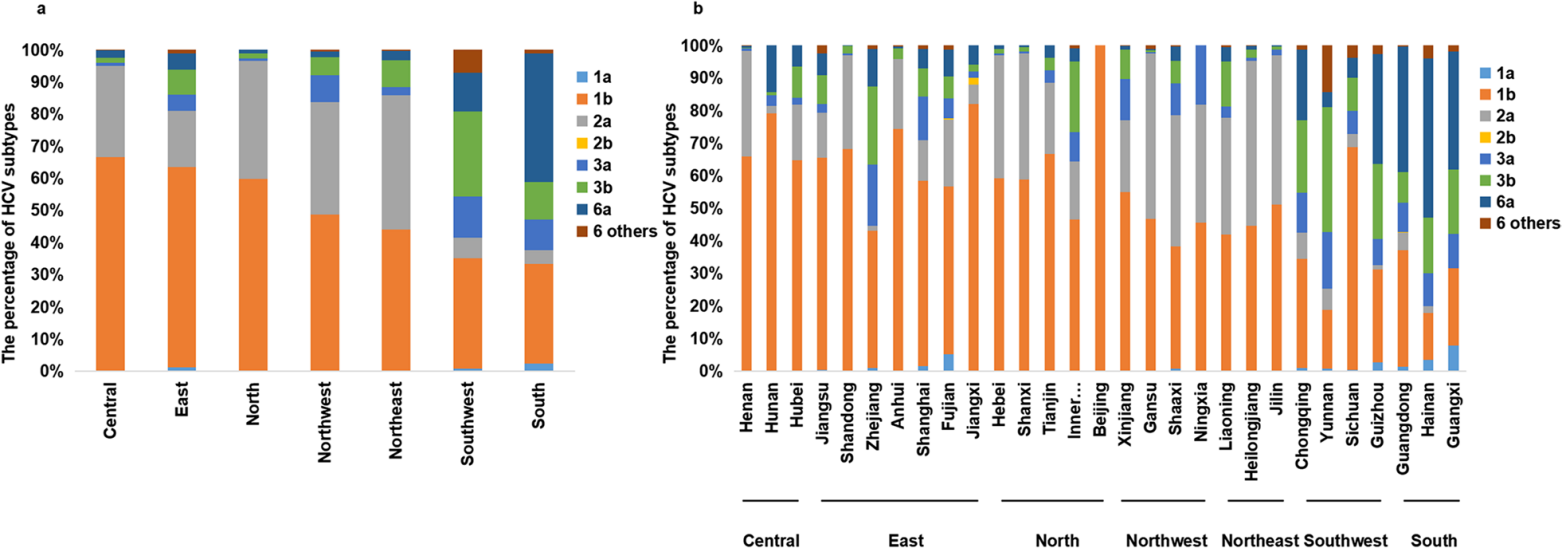
The distribution of HCV subtypes in diverse regions in Chinese mainland. **a** and **b** shows the distribution of HCV subtypes in seven regions of Chinese mainland and diverse provinces/municipalities, respectively. In **a**, the horizontal axis represents diverse regions of Chinese mainland, and the vertical axis represents the percentage of HCV subtypes. In **b**, the horizontal axis represents diverse provinces/municipalities, and the vertical axis represents the percentage of HCV subtypes. The data in this figure are consistent with those in Table 1

The distribution of the HCV subtypes varied in the different regions. Figure 3a and b show the distribution of HCV subtypes in seven regions of Chinese mainland (north, northwest, northeast, central, east, south and southwest) and diverse provinces/municipalities, respectively. Overall, subtype 1b was dominant in Chinese mainland, except for subtype 6a, which had a significant prevalence in the southern region. Subtype 2a was the second most common subtype in most regions, including the northern, northwestern, and northeastern regions and central China. However, subtype 3b was second most common in the southwestern region, and subtype 1b was second most common in the southern region.

Specifically, in central China, subtype 1b was dominant, accounting for 66.6%, followed by subtype 2a (28.3%), 6a (2.3%), subtype 3b (1.6%) and subtype 3a (1.1%). In the eastern region, subtype 1b was dominant, accounting for 62.4%, followed by subtype 2a (17.4%), 3b (7.8%), 3a (5.1%), 6a (5.0%), 1a (1.1%) and other rare subtypes of genotype 6 (1.1%). In the northern region, subtype 1b was dominant, accounting for 59.5%, followed by subtypes 2a (36.8%), 3b (1.5%), 6a (1.2%) and 3a (0.8%). In the northwestern region, subtype 1b was dominant, accounting for 48.5%, followed by subtypes 2a (35.2%), 3a (8.3%), 3b (5.8%) and 6a (1.6%). In the northeastern region, subtype 1b was dominant, accounting for 44.0%, followed by subtypes 2a (41.8%), 3b (8.4%), 6a (2.8%) and 3a (2.5%). In the southwestern region, subtype 1b was dominant, accounting for 34.2%, followed by subtypes 3b (26.4%), 3a (13.0%), 6a (12.0%), and other rare subtypes of genotypes 6 (7.2%) and 2a (6.3%). In the southern region, subtype 6a was dominant, accounting for 40.0%, followed by subtype 1b (30.9%), 3b (11.8%), 3a (9.4%), 2a (4.3%), 1a (2.4%) and other rare subtypes among genotype 6 (1.2%). The more detailed description of the distribution of subtypes in diverse regions and provinces/municipalities are shown in Table 1.

**Table 1:** The distribution of HCV subtypes in diverse regions and provinces/municipalities/autonomous regions in Chinese Mainland

Area	Number	HCV subtype, %							
		1a	1b	2a	2b	3a	3b	6a	6 others
**Central**	**1402**	**0**	***66.6***	**28.3**	**0**	**1.1**	**1.6**	**2.3**	**0.1**
Henan	1124	0	65.9	32.3	0	0.8	0.3	0.6	0.1
Hunan	91	0	79.1	2.2	0	3.3	1.1	14.3	0
Hubei	187	0	64.7	17.1	0	2.1	9.6	6.4	0
**East**	**1787**	**1.1**	***62.4***	**17.4**	**0.1**	**5.1**	**7.8**	**5.0**	**1.1**
Jiangsu	492	0.4	65.0	13.8	0	2.6	8.9	6.7	2.4
Shandong	403	0	68.2	28.8	0	0.5	2.2	0.2	0
Zhejiang	191	1.0	41.9	1.6	0	18.8	24.1	11.5	1.0
Anhui	238	0	74.4	21.4	0	0	3.4	0.4	0.4
Shanghai	185	1.6	56.8	12.4	0	13.5	8.6	5.9	1.1
Fujian	228	5.3	51.3	20.6	0.4	6.1	6.6	8.3	1.3
Jiangxi	50	0	82.0	6.0	2.0	2.0	2.0	6.0	0
**North**	**1346**	**0.2**	***59.5***	**36.8**	**0**	**0.8**	**1.5**	**1.2**	**0**
Hebei	956	0.2	59	37.7	0	0.6	1.4	1.2	0
Shanxi	209	0	58.9	38.8	0	0.5	1.4	0.5	0
Tianjin	78	0	66.7	21.8	0	3.8	3.8	3.8	0
Inner Mongolia	101	0	46.5	17.8	0	8.9	21.8	4.0	1.0
Beijing	2	0	100	0	0	0	0	0	0
**Northwest**	**1075**	**0.2**	***48.5***	**35.2**	**0**	**8.3**	**5.8**	**1.6**	**0.6**
Xinjiang	497	0	54.9	22.1	0	12.7	8.9	1.2	0.2
Gansu	334	0	46.7	50.9	0	0.3	0.6	0.3	1.2
Shaaxi	233	0.9	37.3	40.3	0	9.9	6.9	4.3	0.4
Ningxia	11.0	0	45.5	36.4	0	18.2	0	0	0
**Northeast**	**1553**	**0**	***44.0***	**41.8**	**0**	**2.5**	**8.4**	**2.8**	**0.4**
Liaoning	855	0	41.9	35.9	0	3.5	13.7	4.4	0.6
Heilongjiang	473	0	44.6	50.5	0	1.1	2.5	1.1	0.2
Jilin	225	0	51.1	45.8	0	1.8	0.9	0.4	0
**Southwest**	**1955**	**0.9**	***34.2***	**6.3**	**0**	**13.0**	**26.4**	**12.0**	**7.2**
Chongqing	689	1.0	33.4	8.1	0	12.2	22.4	21.6	1.3
Yunnan	789	0.8	18.1	6.5	0	17.2	38.5	4.4	14.4
Sichuan	403	0.5	68.2	4.0	0	7.2	10.2	6.2	3.7
Guizhou	74	2.7	28.4	1.4	0	8.1	23	33.8	2.7
**South**	**1633**	**2.4**	**30.9**	**4.3**	**0.1**	**9.4**	**11.8**	***40.0***	**1.2**
Guangdong	1201	1.4	35.6	5.4	0.1	9.1	9.5	38.5	0.4
Hainan	280	3.6	14.3	2.1	0	10.0	17.1	48.9	3.9
Guangxi	152	7.9	23.7	0	0	10.5	19.7	36.2	2.0

The proportion of subtypes in diverse regions is shown in bold. The highest proportion of subtype in diverse regions is shown in bold italics

#### Geographical distribution of each HCV subtype

The geographical distribution of each subtype in diverse regions is shown in Table 2. The geographical distribution of each subtype in diverse provinces/municipalities is shown in Additional file 3: Table S1.

**Table 2:** The geographical distribution of each subtype in diverse regions in Chinese Mainland

Subtype	Region, %						
	North	Northwest	Northeast	Central	East	Southwest	South
1b	15.1	10.0	13.1	17.9	***21.4***	12.8	9.7
2a	19.8	15.7	***27.0***	16.5	12.9	5.2	3.0
3a	2.9	13.4	5.9	2.4	13.7	***38.5***	23.1
3b	3.7	5.6	11.9	2.0	12.6	***46.8***	17.4
6a	1.7	1.6	4.0	2.9	8.3	21.5	***60.0***
6 others	0.5	3.1	3.1	0.5	10.4	***72.5***	9.8

The highest geographical distribution of each subtype in diverse regions is shown in bold italics

HCV subtype 1b was found most frequently in the eastern region (21.4%) and gradually decreased from central China (17.9%) to the northern region (15.1%), northeastern region (13.1%) and northwestern region (10.0%). The lowest prevalence was found in the southern region (9.7%).

The prevalence of HCV subtype 2a gradually decreased from northern parts of Chinese mainland to southern parts of Chinese mainland. The percentage of subtype 2a was highest in northern parts of Chinese mainland, 27.0% in the northeastern region, 19.8% in the northern region and 15.7% in the northwestern region, with a lower prevalence in the southwestern region (5.2%) and southern region (3.0%).

The prevalence of HCV subtype 3a gradually increased from northern parts of Chinese mainland to southern parts of Chinese mainland, showing a trend similar to that of subtype 3b, which was opposite to the prevalence of subtype 2a. Both subtypes 3a and 3b were found most frequently in the southwestern region, were highly prevalent in southern region and had the lowest prevalence in central China. For subtype 3a, the prevalence was as high as 38.5% in the southwestern region, followed by the southern, eastern, northwestern, northeastern and northern regions, which had prevalence of 23.1%, 13.7%, 13.4% 5.9%, and 2.9%, respectively. For subtype 3b, the prevalence was as high as 46.8% in the southwestern region, followed by the southern, eastern, northeastern, northwestern and northern regions, which had prevalence of 17.4%, 12.6%, 11.9% and 5.6% and 3.7%, respectively. The prevalence of subtypes 3a (2.4%) and 3b (2.0%) was lowest in central China.

Subtype 6a was most prevalent in the southern region (60.0%), especially in Guangdong Province (42.4%), followed by the southwestern and eastern regions, which had prevalence of 21.5% and 8.3%, respectively. Other rare subtypes among genotype 6 were most prevalent in the southwestern region (72.5%), especially in Yunnan Province (59.1%).

#### Population distribution of HCV subtypes

**Fig. 4:**
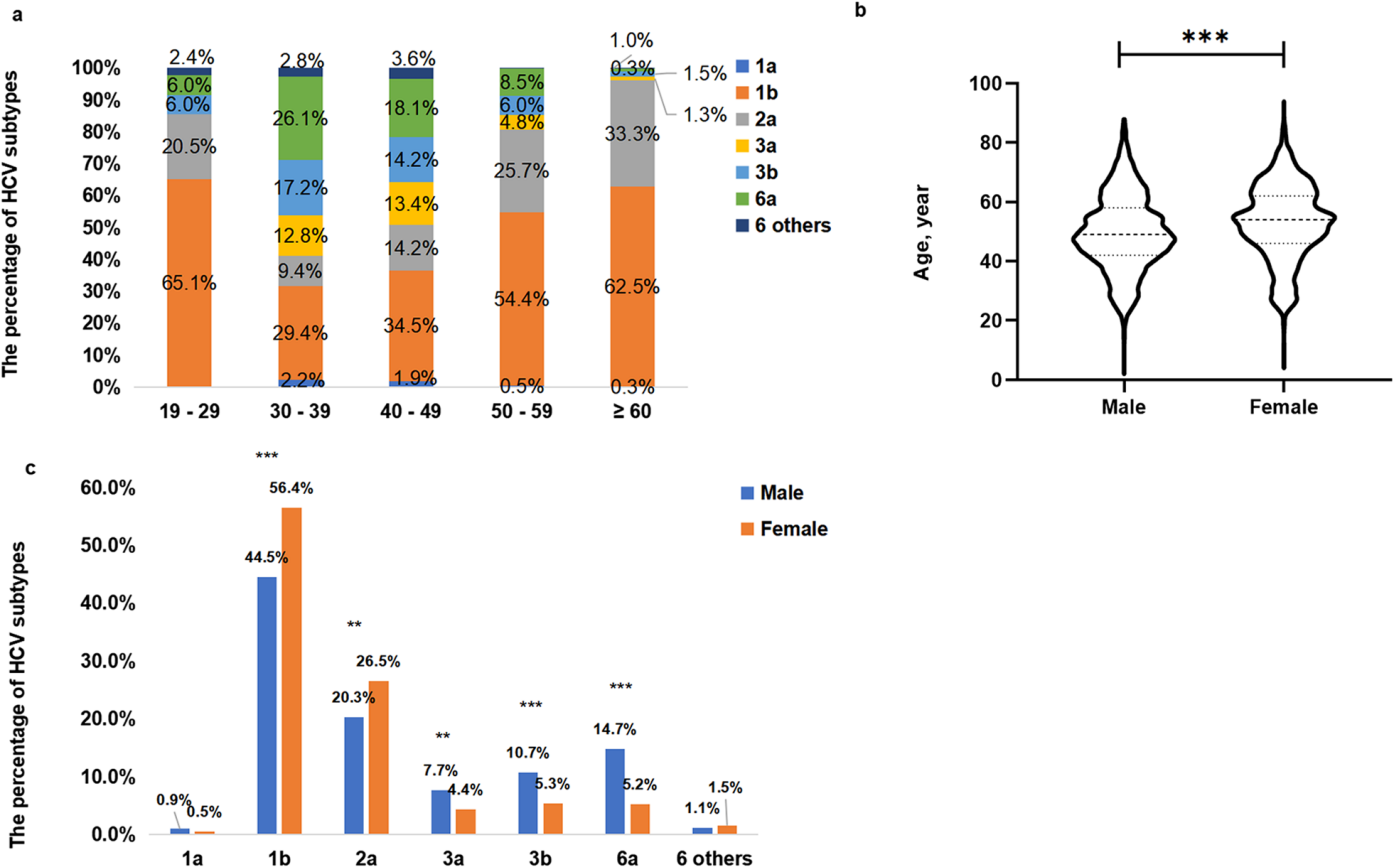
The distribution of HCV subtypes among different age groups. The horizontal axis represents different age groups (19–29, 30–39, 40–49, 50–59, ≥ 60 year), and the vertical axis represents the percentage of different subtypes (**a**). **b** compares the age of HCV-infected patients in different sexes. The horizontal axis represents different sexes, and the vertical axis represents age. **c** shows the proportions of subtypes in different sexes. The horizontal axis represents diverse HCV subtypes, and the vertical axis represents the percentage of diverse HCV subtypes in different sexes. *P* < 0.01 is marked by ** and *P* < 0.001 is marked by ***

The data in Fig. 4a show the distribution of subtypes among different age groups, and the mean age of the HCV carriers was 51 years old. Subtype 1b had the highest proportion among each group, especially in carriers aged 19 to 29 years old. A relatively lower proportion of subtype 1b and higher proportions of genotypes 3 and 6 were found in HCV carriers aged 30 to 49 years old than in other age groups. The mean age of female carriers was significantly older than that of male carriers (53 versus 50, *P* < 0.001) (Fig. 4b). The data in Fig. 4c show that male carriers had higher proportions of subtypes 3a, 3b, 6a, and other rare subtypes among genotype 6 and lower proportions of subtypes 1b and 2a than female carriers (*P* < 0.01).

#### HCV subtypes and viral load

The mean viral loads of subtypes 1a, 1b, 2a, 3a, 3b, 6a and other rare subtypes among genotype 6 were 4.1E + 06, 3.8E + 06, 9.6E + 05, 4.0E + 06, 3.8E + 06, 1.4E + 06 and 6.7E + 06 IU/ml (Table 3). The highest mean viral load was found in other rare subtypes among genotype 6, followed by subtypes 1a, 3a, 1b, 3b, 6a and subtype 2a. For most subtypes, more than 70% of carriers had a viral load concentrated in the range from 1.0E + 05 to 1.0E + 07 IU/ml, except for subtype 2a and other rare subtypes among genotype 6. Moreover, the highest proportion of viral load was concentrated in the range from 1.0E + 06 to 1.0E + 07 IU/ml in most subtypes but not subtype 2a, which was concentrated in the range from 1.0E + 05 to 1.0E + 06 IU/ml.

**Table 3:** Viral load among each HCV subtype

Subtype (number)	Viral load (Log_10_, IU/ml)						Mean, IU/ml
	< 3	3–4	4–5	5–6	6–7	7–8	
1a (*n* = 70)	0.0%	7.1%	7.1%	34.3%	***35.7%***	15.7%	4.1E + 06
1b (*n* = 3730)	0.2%	3.9%	7.6%	26.0%	***53.0%***	9.3%	3.8E + 06
2a (*n* = 1787)	0.6%	12.9%	23.8%	***36.1%***	26.0%	0.6%	9.6E + 05
3a (*n* = 502)	0.0%	4.0%	10.4%	27.1%	***46.8%***	11.8%	4.0E + 06
3b (*n* = 820)	0.0%	2.3%	9.6%	30.4%	***47.6%***	10.1%	3.8E + 06
6a (*n* = 727)	0.3%	7.6%	16.5%	32.6%	***42.4%***	0.7%	1.4E + 06
6 others* (*n* = 157)	0.0%	4.5%	8.3%	22.3%	***44.6%***	20.4%	6.7E + 06

*6 others include subtypes 6n, 6k, 6u, 6g 6v 6e and 6c. The highest distribution of viral load among each subtype is shown in bold italics

### Phylogenetic analysis

**Fig. 5:**
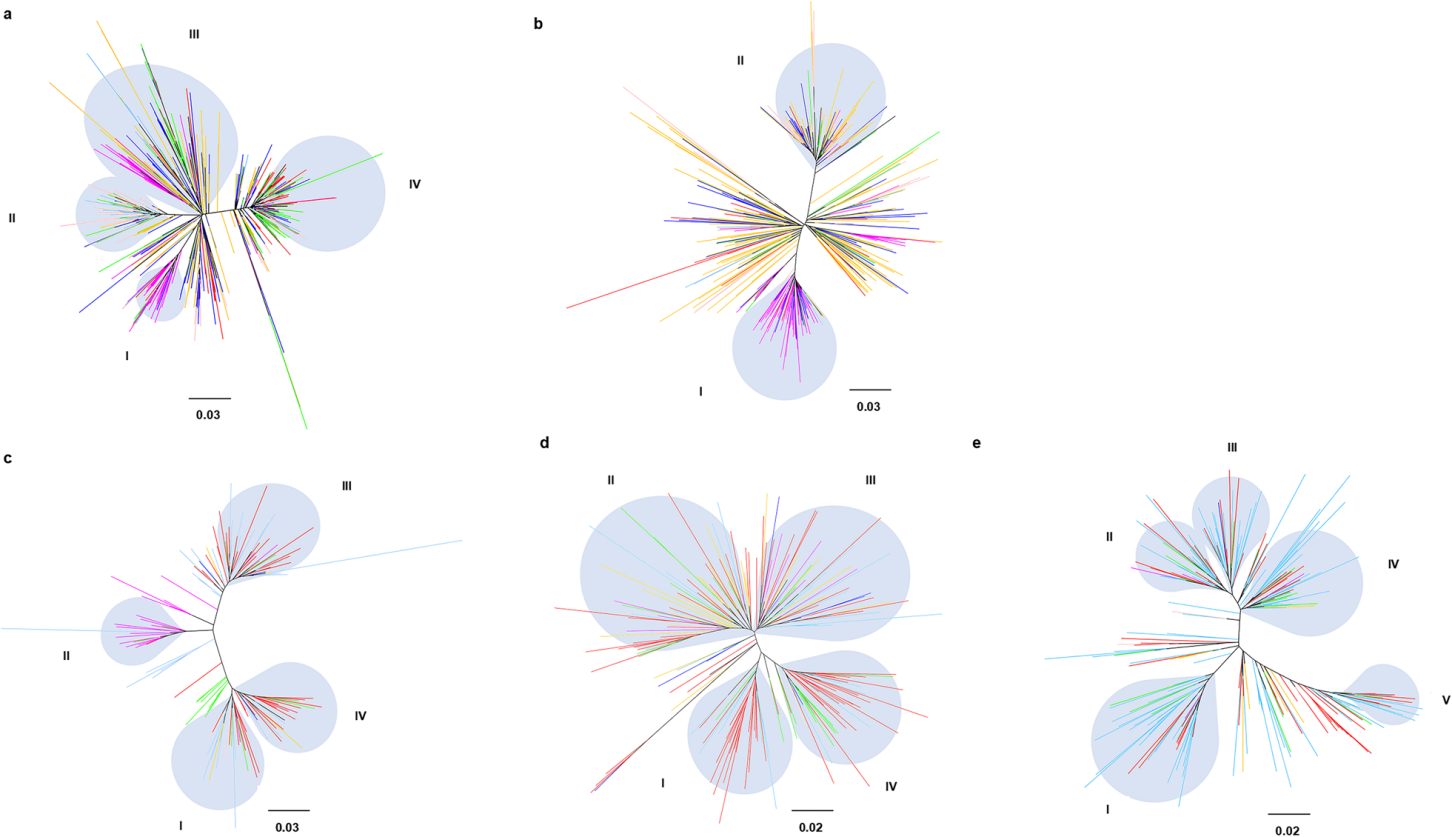
Phylogenetic analysis of each HCV subtype. Maximum likelihood trees were constructed by NS5B partial sequences. **a**–**e** shows the phylogenetic analysis results of HCV subtypes 1b, 2a, 3a, 3b and 6a. Maximum likelihood trees are presented by radial tree layout. Regions are specified according to color.  Southern region  Southwestern region  Northwestern region  Northern region  Eastern region  Northeastern region  Central China. The bar below the figure shows the scale for nucleotide substitutions per site

A total of 2044 sequences from 142 districts and counties in 28 provinces and municipalities were available for phylogenic analysis, containing subtypes 1b (*n* = 993), 2a (*n* = 471), 3a (*n* = 136), 3b (*n* = 198) and 6a (*n* = 246). The proportion of each subtype that we used for phylogenic analysis was between 18.0% and 22.6% of the overall. Additional file 3: Table S2 shows the distribution of subtypes used for phylogenic analysis. We performed phylogenetic analysis of each subtype based on NS5B sequences to explore the genetic relationship between sequences from diverse regions of Chinese mainland (Fig. 5 and Additional file 2: Fig. S2).

For subtype 1b (Fig. 5a), the sequences were mainly divided into four clusters, and most sequences were from northern parts of Chinese mainland (including the northern, northeastern and northwestern regions). Cluster I mainly contained sequences from northwestern region, while clusters II, III and IV mainly contained sequences from central China and the southern region, northern parts of Chinese mainland, and the southwestern and eastern regions, respectively. Additionally, the phylogenetic analysis showed that most sequences from the northern, northwestern and northeastern regions were clustered, indicating a close genetic relationship between sequences from these regions. Most sequences from central China were clustered with sequences from the southern and northern regions. Most sequences from the eastern were clustered with sequences from central China and the northeastern region. Most sequences from the southwestern region were clustered with sequences from the northwestern region and central China. Most sequences from the southern region were clustered with sequences from central China. These results indicate that sequences with close genetic relationships are generally geographically bordering.

For subtype 2a (Fig. 5b), there were two clusters, and most sequences were from northern parts of Chinese mainland. Cluster I mainly contained sequences from the northwestern region, and cluster II mainly contained sequences from the northeastern and northern regions. The phylogenetic analysis showed that most sequences from the northern, northwestern and northeastern regions were clustered, indicating a close genetic relationship between sequences from these regions. Most sequences from central China were clustered with sequences from the northern parts of Chinese mainland. For subtype 3a (Fig. 5c), most sequences were from the southwestern and southern regions, and fewer sequences were from the northwestern and eastern regions. There were four clusters: both Clusters I and II mainly contained sequences from the southwestern and southern regions, while sequences from the northwestern region were concentrated in Cluster I, and cluster IV mainly contained sequences from the southwestern region. Additionally, the phylogenetic analysis showed that most sequences from other regions were clustered with sequences from the southern and southwestern regions, indicating that the national spread of subtype 3a was associated with sequences from the southwestern and southern regions.

For subtype 3b (Fig. 5d), most sequences were from the southwestern and southern regions, and fewer sequences were from the northern parts of Chinese mainland. There were four clusters; both Clusters I and IV mainly contained sequences from the southwestern region, cluster II mainly contained sequences from the southwestern and northeastern regions, and cluster III mainly contained sequences from the northern parts of Chinese mainland and southwestern region. The main result of phylogenetic analysis was that most sequences from the southwestern region were clustered with sequences from the southern region. Most sequences from the eastern and northeastern regions were clustered with sequences from the southwestern region, indicating that the national spread of subtype 3b was associated with sequences from the southwestern and southern regions.

For subtype 6a (Fig. 5e), most sequences were from the southern and southwestern regions, and fewer sequences were from the eastern region. There were five clusters: Cluster I mainly contained sequences from the southwestern, southern and eastern regions, and clusters II to V mainly contained sequences from the southwestern and southern regions. The phylogenetic analysis showed that most sequences from the southwestern and eastern regions were clustered with sequences from the southern region, suggesting a close genetic relationship of these sequences.

## Discussion

In the present study, investigation of the geographical and population distribution of HCV genotypes showed that the distribution varied in different populations and geographic regions in Chinese mainland. Our data were closer to the true distribution of HCV genotypes as more patients were diagnosed with HCV infection [20]. The top five subtypes were subtypes 1b (48.5%), 2a (22.4%), 3b (10.3%), 6a (10.1%) and 3a (6.2%), and genotypes 4, 5, 7 and 8 were not found, which is similar to the data from a previous study carried out in 2017. Moreover, both studies revealed that the prevalence of subtypes 1a and 2b was rare, less than 1% [17]. The difference is that the proportion of HCV subtypes was changed and genetic diversity increased. The proportion of subtypes 1b and 2a decreased, while 3b, 3a, 6a and other rare subtypes among genotype 6 increased compared to a previous study, which may be explained by changes in transmission, socioeconomic advancement migration and improvement of screening and diagnosis of HCV infection [19, 20]. Additionally, another study indicated that the proportion of genotypes 3 and 6 gradually increased from 1993 to 2017, especially in southern regions [23]. Moreover, genotype 3, especially subtype 3b, obtained a relatively lower sustained virological response than the other genotypes [9, 24, 25]. Therefore, we should pay more attention to genotype 3. Our data revealed the increasing trend of genotype 3, especially in southern parts of Chinese mainland, which provided a basis for strategies of HCV management, thus contributing to the goals to eliminate viral hepatitis by 2030.

The proportion of subtypes varied in different regions in Chinese mainland. Our data showed more diversity of subtypes and a higher prevalence of genotypes 3 and 6 in southern parts of Chinese mainland than in northern parts of Chinese mainland. To some extent, this may be related to the unique geographical conditions of southern parts of Chinese mainland, which bordered south and southeast Asia, where the predominant genotypes were 3 and 6, respectively [16]. Our previous research found a close genetic relationship between genotype 3-infected patients from southwestern and southern regions of Chinese mainland and patients from Vietnam [26]. Additionally, other studies indicated that genotype 6a in Guangdong Province and genotype 6xg in Yunnan originated from Vietnam and Myanmar, respectively [27, 28]. In addition, the southwestern region of Chinese mainland is vital for drug smuggling, and infections of genotypes 3 and 6 are mainly transmitted by intravenous drug abuse [29, 30]. Thus, these may explain genotypes 3 and 6 were most common in southern parts of Chinese mainland. Northern region of Chinese mainland bordered eastern Europe, where genotypes 1 and 3 were two major genotypes. The northeastern region of Chinese mainland neighbors the high-income Asia Pacific (the Republic of Korea and Japan), where the major genotypes were genotypes 1 and 2 [16]. Thus, this may explain the high prevalence of genotypes 1 and 2 in northern parts of Chinese mainland.

Moreover, our data showed that the distribution of subtypes in diverse regions was different, which was not investigated by previous studies [17, 18]. Subtype 1b was most prevalent in the eastern region, and subtype 2a was most prevalent in the northeastern region. Subtypes 3a and 3b and other rare subtypes among genotype 6 were most prevalent in the southwestern region, and subtype 6a was most prevalent in the southern region. Regarding province, subtypes 1b and 2a were most prevalent in Henan Province, subtypes 3a and 3b and the rare subtype among genotype 6 were most prevalent in Yunnan Province, and subtype 6a was most prevalent in Guangdong Province. The result was slightly different from the result from a previous study, which indicated that genotypes 1, 2, 3 and 6 were most prevalent in Jiangsu, Jilin, Yunnan and Hainan provinces, respectively [17]. Notably, the prevalence of subtype 6n was higher than that of subtype 6a in Yunnan Province, which is in contrast to the general distribution pattern in other areas.

Additionally, the distributions of subtypes were different in terms of age and sex. Our data showed that the proportions of genotypes 3 and 6 were much higher in patients aged from 30 to 50 years, showing more diversity of subtypes. Additionally, the proportion of subtypes by sex was different. Apart from these, we found that females with HCV infection were older than males, which is in accordance with the results of a previous study [17]. The different distribution of subtypes in populations may be explained by the different behaviors and transmission. In addition, among the patients who were diagnosed with HCV infection for the first time, 58.1% were over 50 years old, and 84.4% were over 40 years old. Therefore, we should pay more attention to the older age population when screening for HCV infection, which helps to optimize strategies for the management of HCV infection to improve the diagnosis rate.

The phylogenetic analysis for each subtype indicated a different molecular transmission among each subtype. Both subtypes 1b and 2a were mainly prevalent in northern parts of Chinese mainland, and the close evolutionary relationship of sequences from these regions indicates that they may have the same ancestor. Then, there was a national spread from northern parts to southern parts of Chinese mainland. Both subtypes 3a and 3b were mainly prevalent in southern parts of Chinese mainland, and the phylogenetic analysis indicated that sequences from southern parts of Chinese mainland may have the same ancestor. Then, there was a national spread from southern parts to northern parts of Chinese mainland. Moreover, subtype 6a was mainly prevalent in the southern region and spread to other regions.

There are some limitations of our study. First, we did not use a multistage stratified sampling method, so the findings on changes in genotype proportions may be influenced by sample selection. However, we enrolled a large number of patients covering 29 provinces/municipalities, 207 districts and counties in Chinese mainland, which could improve the representativeness of our data. Second, 257 patients failed to genotype, probably because the viral load was too low to be successfully detected. Another reason may be unidentified genotype infection that needs to be further explored.

## Conclusions

HCV subtypes 1b and 2a remained the most common subtypes in Chinese mainland, but their proportions decreased over the past years, while the proportions of genotypes 3 and 6 increased. Our investigation of the geographic and population distributions of HCV subtypes and the phylogenetic analysis of each subtype provided an accurate epidemiological picture of the circulating viral strains in Chinese mainland. It is vital to the prevention, diagnosis and treatment of HCV infection and finally contributes to eliminating viral hepatitis as proposed by the WHO.

## Supplementary Information

**Additional file 1: Fig. S1.** The proportion of enrolled patients to the total population of the province/municipality. Data on the total population of each province/municipality are available at http://www.stats.gov.cn/. The horizontal axis represents diverse provinces/municipalities, and the vertical axis represents the proportion of enrolled patients to the total population of the province/municipality.

**Additional file 2: Fig. S2.** Phylogenetic analysis of each HCV subtype. Maximum likelihood trees were constructed by NS5B partial sequences. a-e shows the phylogenetic analysis results of HCV subtypes 1b, 2a, 3a, 3b and 6a. These figures were presented by polar tree layout and generated by the same dataset as Fig. 5. Regions are specified according to the same color as in Fig. 5.

**Additional file 3: Table S1.** The geographical distribution of each subtype in diverse provinces/municipalities. **Table S2**. The geographical distribution of subtypes used for phylogenic analysis.

## Data Availability

The datasets used and analyzed during the current study are available from the corresponding author on reasonable request.

## Abbreviations

DAA: Direct antiviral agents
GTR: General–time–reversible
HCV: Hepatitis C virus
ML: Maximum likelihood
PCR: Polymerase chain reaction
RNA: Ribonucleic acid
TIB: Triplex International Biosciences
WHO: World Health Organization

## References

[1] Lee MH, Yang HI, Lu SN, Jen CL, You SL, Wang LY (2012). Chronic hepatitis C virus infection increases mortality from hepatic and extrahepatic diseases: a community-based long-term prospective study. J Infect Dis.

[2] Backus LI, Boothroyd DB, Phillips BR, Belperio P, Halloran J, Mole LA (2011). A sustained virologic response reduces risk of all-cause mortality in patients with hepatitis C. Clin Gastroenterol Hepatol.

[3] Blach S, Terrault NA, Tacke F, Gamkrelidze I, Craxi A, Tanaka J (2022). Global change in hepatitis C virus prevalence and cascade of care between 2015 and 2020: a modelling study. Lancet Gastroenterol Hepatol.

[4] Chinese Center for Disease Control and Prevention. The status of notifiable infectious diseases in China in 2020. http://www.nhc.gov.cn/jkj/s3578/202103/f1a448b7df7d4760976fea6d55834966.shtml. Accessed 12 Sept 2021 (in Chinese).

[5] WHO. Combating hepatitis B and C to reach elimination by 2030. Geneva, Switzerland: World Health Organization, 2016.

[6] WHO. Interim guidance for country validation of viral hepatitis elimination. Geneva, Switzerland: World Health Organization, 2021.

[7] Lee MH, Hsiao TI, Subramaniam SR, Le AK, Vu VD, Trinh HN (2017). HCV Genotype 6 increased the risk for hepatocellular carcinoma among Asian patients with liver cirrhosis. Am J Gastroenterol.

[8] Kanwal F, Kramer JR, Ilyas J, Duan Z, El-Serag HB (2014). HCV genotype 3 is associated with an increased risk of cirrhosis and hepatocellular cancer in a national sample of US Veterans with HCV. Hepatology.

[9] Blanco JR, Rivero-Juarez A (2016). HCV genotype 3: a wolf in sheep's clothing. Expert Rev Anti Infect Ther.

[10] Liu X, Hu P (2021). Efficacy and safety of glecaprevir/pibrentasvir in patients with chronic HCV infection. J Clin Transl Hepatol.

[11] Tang Q, Wei L, Liu X, Hu P (2021). Sofosbuvir-based therapies achieved satisfactory virological response in Chinese individuals with genotypes 3 and 6 infections: a real-world experience. Infect Drug Resist.

[12] Chinese Society of H, Chinese Society of Infectious Diseases CMA (2019). [Guidelines for the prevention and treatment of hepatitis C(2019 version)]. Zhonghua Gan Zang Bing Za Zhi.

[13] European Association for the Study of the Liver. Electronic address eee, Clinical Practice Guidelines Panel C, representative EGB, Panel m (2020). EASL recommendations on treatment of hepatitis C: Final update of the series(). J Hepatol.

[14] Panel A-IHG (2018). Hepatitis C Guidance 2018 Update: AASLD-IDSA recommendations for testing, managing, and treating hepatitis C virus infection. Clin Infect Dis.

[15] Messina JP, Humphreys I, Flaxman A, Brown A, Cooke GS, Pybus OG (2015). Global distribution and prevalence of hepatitis C virus genotypes. Hepatology.

[16] Blach S, Zeuzem S, Manns M, Altraif I, Duberg A-S, Muljono DH (2017). Global prevalence and genotype distribution of hepatitis C virus infection in 2015: a modelling study. Lancet Gastroenterol Hepatol.

[17] Chen Y, Yu C, Yin X, Guo X, Wu S, Hou J (2017). Hepatitis C virus genotypes and subtypes circulating in Mainland China. Emerg Microbes Infect.

[18] Ju W, Yang S, Feng S, Wang Q, Liu S, Xing H (2015). Hepatitis C virus genotype and subtype distribution in Chinese chronic hepatitis C patients: nationwide spread of HCV genotypes 3 and 6. Virol J.

[19] Li M, Zhuang H, Wei L (2019). How would China achieve WHO's target of eliminating HCV by 2030?. Expert Rev Anti Infect Ther.

[20] Chinese Center for Disease Control and Prevention. The status of notifiable infectious diseases in China. http://www.nhc.gov.cn/jkj/s3578/201802/de926bdb046749abb7b0a8e23d929104.shtml. Accessed 20 Mar 2023 (in Chinese).

[21] Yang Z (1994). Estimating the pattern of nucleotide substitution. J Mol Evol.

[22] Price MN, Dehal PS, Arkin AP (2009). FastTree: computing large minimum evolution trees with profiles instead of a distance matrix. Mol Biol Evol.

[23] Thrift AP, El-Serag HB, Kanwal F (2017). Global epidemiology and burden of HCV infection and HCV-related disease. Nat Rev Gastroenterol Hepatol.

[24] Du G, Li X, Musa TH, Ji Y, Wu B, He Y (2019). The nationwide distribution and trends of hepatitis C virus genotypes in mainland China. J Med Virol.

[25] Leroy V, Angus P, Bronowicki JP, Dore GJ, Hezode C, Pianko S (2016). Daclatasvir, sofosbuvir, and ribavirin for hepatitis C virus genotype 3 and advanced liver disease: a randomized phase III study (ALLY-3+). Hepatology.

[26] Huang R, Rao H, Xie Q, Gao Z, Li W, Jiang D (2019). Comparison of the efficacy of sofosbuvir plus ribavirin in Chinese patients with genotype 3a or 3b HCV infection. J Med Virol.

[27] Liu X, Chen Z, Tang Q, Hu P (2022). Phylogenetic signature and prevalence of natural resistance-associated substitutions for hepatitis C virus genotypes 3a and 3b in southwestern China. J Virus Erad.

[28] Xu R, Wang H, Huang J, Wang M, Liao Q, Shan Z (2022). Complete genome sequencing and evolutionary analysis of hepatitis C virus subtype 6a, including strains from Guangdong Province. China Arch Virol.

[29] Chen M, Ma Y, Chen H, Dai J, Luo H, Jia M (2019). Complete genome sequencing and evolutionary analysis of HCV subtype 6xg from IDUs in Yunnan, China. PLoS ONE.

[30] Ruta S, Cernescu C (2015). Injecting drug use: a vector for the introduction of new hepatitis C virus genotypes. World J Gastroenterol.

