# Supplementary figures and images for: Molecular epidemiology of hepatitis C virus genotypes in different geographical regions of Chinese mainland and a phylogenetic analysis
Source: Infect Dis Poverty. 2023 Jul 10;12:66. doi: 10.1186/s40249-023-01106-y (PMC10331966; doi:10.1186/s40249-023-01106-y)

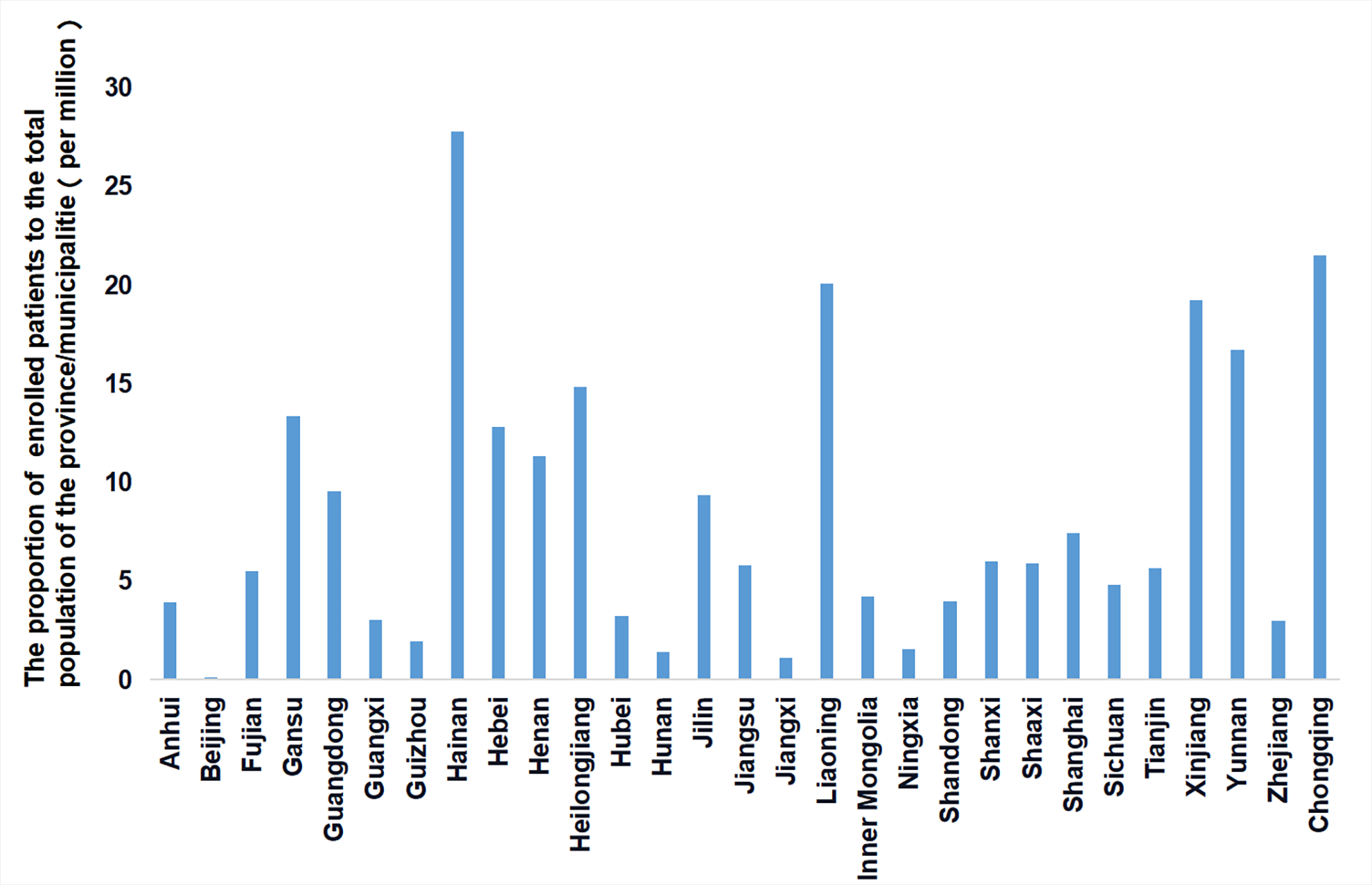

Supplement: Supplementary file 1 — Additional file 1: Fig. S1. The proportion of enrolled patients to the total population of the province/municipality. Data on the total population of each province/municipality are available at http://www.stats.gov.cn/. The horizontal axis represents diverse provinces/municipalities, and the vertical axis represents the proportion of enrolled patients to the total population of the province/municipality. [file 40249_2023_1106_MOESM1_ESM.tif]

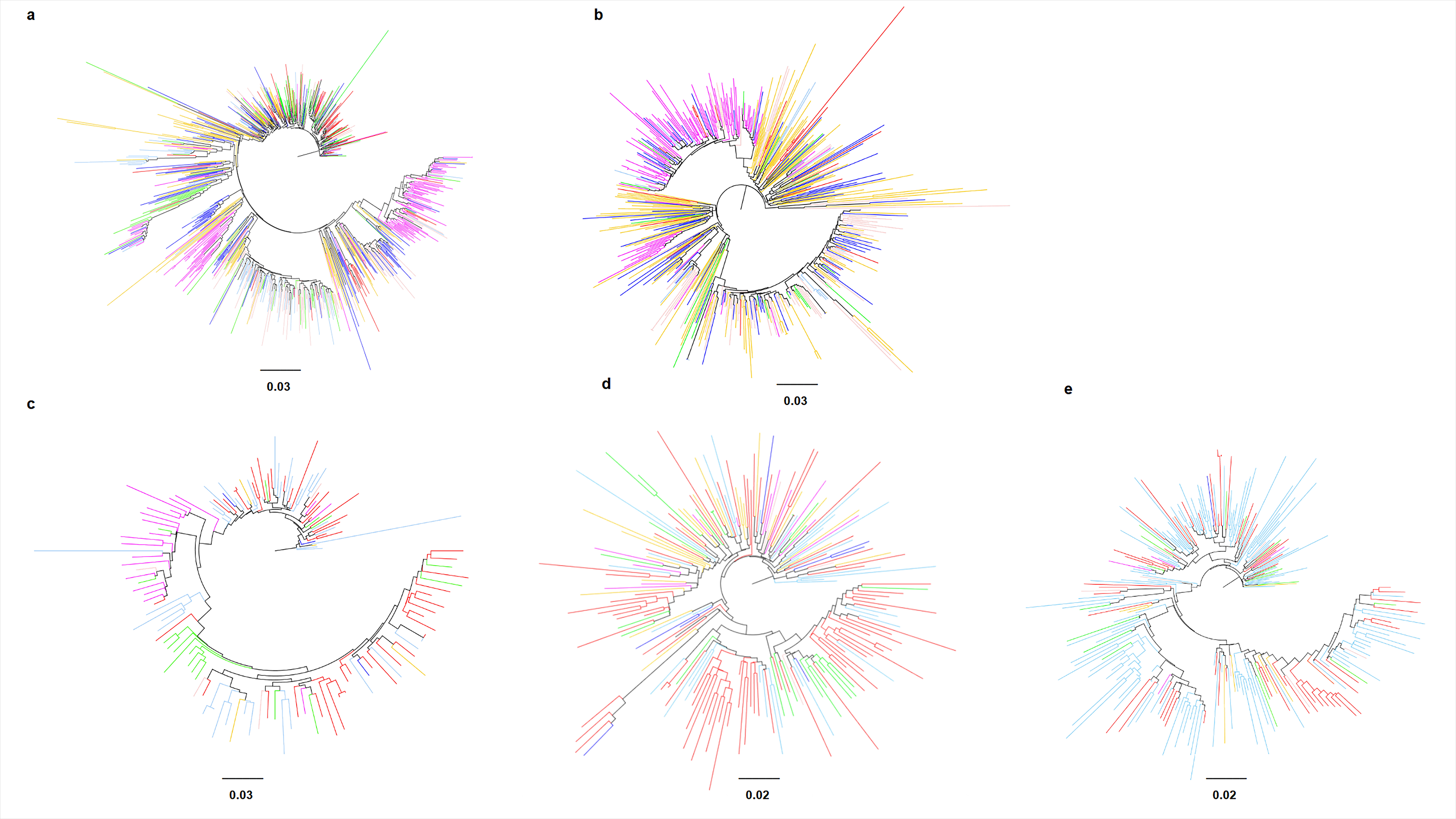

Supplement: Supplementary file 2 — Additional file 2: Fig. S2. Phylogenetic analysis of each HCV subtype. Maximum likelihood trees were constructed by NS5B partial sequences. a-e shows the phylogenetic analysis results of HCV subtypes 1b, 2a, 3a, 3b and 6a. These figures were presented by polar tree layout and generated by the same dataset as Fig. 5. Regions are specified according to the same color as in Fig. 5. [file 40249_2023_1106_MOESM2_ESM.tif]
