# Supplementary material for: Molecular epidemiology of hepatitis C virus genotypes in different geographical regions of Chinese mainland and a phylogenetic analysis
Source: Infect Dis Poverty. 2023 Jul 10;12:66. doi: 10.1186/s40249-023-01106-y (PMC10331966; doi:10.1186/s40249-023-01106-y)
Supplement: Supplementary file 3 — Additional file 3: Table S1. The geographical distribution of each subtype in diverse provinces/municipalities. Table S2. The geographical distribution of subtypes used for phylogenic analysis. [file 40249_2023_1106_MOESM3_ESM.docx]

Supplemental Table 1 **The geographical distribution of each subtype in diverse provinces/municipalities.**

| Province/ | HCV subtype | | | | | | |
| --- | --- | --- | --- | --- | --- | --- | --- |
| municipality, % | 1a | 1b | 2a | 3a | 3b | 6a | 6 others |
| Hebei | 2.5 | 10.8 | 15.0 | 0.9 | 1.2 | 1.0 | 0.0 |
| Shanxi | 0.0 | 2.4 | 3.4 | 0.2 | 0.3 | 0.1 | 0.0 |
| Tianjin | 0.0 | 1.0 | 0.7 | 0.5 | 0.3 | 0.3 | 0.0 |
| Inner Mongolia | 0.0 | 0.9 | 0.8 | 1.4 | 2.0 | 0.4 | 0.5 |
| Beijing | 0.0 | 0.0 | 0.0 | 0.0 | 0.0 | 0.0 | 0.0 |
| Xinjiang | 0.0 | 5.2 | 4.6 | 9.5 | 4.0 | 0.6 | 0.5 |
| Gansu | 0.0 | 3.0 | 7.1 | 0.2 | 0.2 | 0.1 | 2.1 |
| Shaaxi | 2.5 | 1.7 | 3.9 | 3.5 | 1.5 | 0.9 | 0.5 |
| Ningxia | 0.0 | 0.1 | 0.2 | 0.3 | 0.0 | 0.0 | 0.0 |
| Liaoning | 0.0 | 6.9 | 12.8 | 4.5 | 10.6 | 3.5 | 2.6 |
| Heilongjiang | 0.0 | 4.1 | 9.9 | 0.8 | 1.1 | 0.5 | 0.5 |
| Jilin | 0.0 | 2.2 | 4.3 | 0.6 | 0.2 | 0.1 | 0.0 |
| Jiangsu | 2.5 | 6.1 | 2.8 | 2.0 | 4.0 | 3.0 | 6.2 |
| Shandong | 0.0 | 5.3 | 4.8 | 0.3 | 0.8 | 0.1 | 0.0 |
| Zhejiang | 2.5 | 1.5 | 0.1 | 5.4 | 4.2 | 2.0 | 1.0 |
| Anhui | 0.0 | 3.4 | 2.1 | 0.0 | 0.7 | 0.1 | 0.5 |
| Shanghai | 3.8 | 2.0 | 1.0 | 3.8 | 1.5 | 1.0 | 1.0 |
| Fujian | 15.2 | 2.2 | 2.0 | 2.1 | 1.4 | 1.7 | 1.6 |
| Jiangxi | 0.0 | 0.8 | 0.1 | 0.2 | 0.1 | 0.3 | 0.0 |
| Henan | 0.0 | 14.2 | 15.1 | 1.4 | 0.3 | 0.6 | 0.5 |
| Hunan | 0.0 | 1.4 | 0.1 | 0.5 | 0.1 | 1.2 | 0.0 |
| Hubei | 0.0 | 2.3 | 1.3 | 0.6 | 1.6 | 1.1 | 0.0 |
| Chongqing | 8.9 | 4.4 | 2.3 | 12.7 | 14.0 | 13.7 | 4.7 |
| Yunnan | 7.6 | 2.7 | 2.1 | 20.5 | 27.6 | 3.2 | 59.1 |
| Sichuan | 2.5 | 5.3 | 0.7 | 4.4 | 3.7 | 2.3 | 7.8 |
| Guizhou | 2.5 | 0.4 | 0.0 | 0.9 | 1.5 | 2.3 | 1.0 |
| Guangdong | 21.5 | 8.2 | 2.7 | 16.5 | 10.3 | 42.4 | 2.6 |
| Hainan | 15.2 | 0.8 | 0.3 | 4.2 | 4.4 | 12.6 | 5.7 |
| Guangxi | 12.7 | 0.7 | 0.0 | 2.4 | 2.7 | 5.1 | 1.6 |

Note: The darker the red color, the higher the proportion of the province/municipality.

Supplemental Table 2 **The geographical distribution of subtypes used for phylogenic analysis.**

| Region, % | Subtype | | | | |
| --- | --- | --- | --- | --- | --- |
|  | 1b | 2a | 3a | 3b | 6a |
| North | 15.4 | 18.7 | 2.2 | 3.5 | 0.4 |
| Northwest | 13.8 | 20.8 | 14.0 | 6.6 | 1.6 |
| Northeast | 14.7 | 27.6 | 2.9 | 14.1 | 2.9 |
| Central | 20.8 | 18.9 | 2.9 | 1.5 | 2.9 |
| East | 12.8 | 4.0 | 12.5 | 12.1 | 7.3 |
| Southwest | 9.9 | 4.7 | 34.6 | 42.9 | 26.0 |
| South | 12.7 | 5.3 | 30.9 | 19.2 | 58.9 |

Note: The darker the red color, the higher the proportion of the region.
